# Supplementary material for: The Effect of a Nucleation Layer on Morphology and Grain Size in MOCVD-Grown β-Ga2O3 Thin Films on C-Plane Sapphire
Source: Materials (Basel). 2022 Nov 24;15(23):8362. doi: 10.3390/ma15238362 (PMC9739698; doi:10.3390/ma15238362)
Supplement: Supplementary file 1 [file materials-15-08362-s001.zip › materials-2043383-supplementary.pdf]

Supplementary Materials

The Effect of a Nucleation Layer on Morphology and Grain Size in MOCVD-Grown β-Ga_2O_3 Thin Films on C-Plane Sapphire

Lauris Dimitrocenko [1], Gundars Strikis [1], Boris Polyakov [1], Liga Bikse [1], Sven Oras [2,3] and Edgars Butanovs [1,*]

[1] Institute of Solid State Physics, University of Latvia, Kengaraga street 8, LV-1063 Riga, Latvia
[2] Institute of Physics, University of Tartu, W. Ostwaldi Str. 1, 50412 Tartu, Estonia
[3] Institute of Technology, University of Tartu, Nooruse 1, 50411 Tartu, Estonia
* Correspondence: edgarsb@cfi.lu.lv

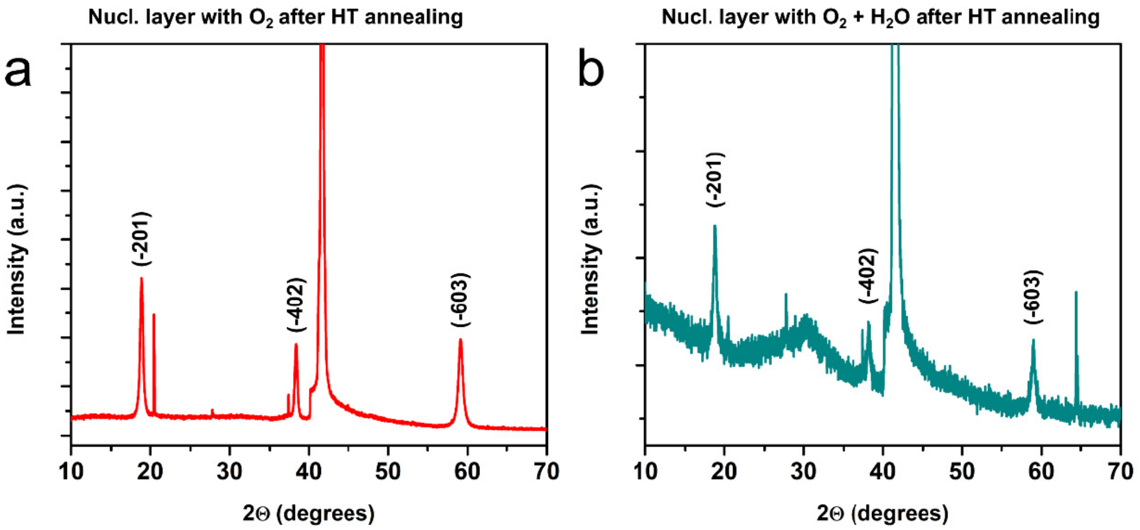

Figure S1. X-ray diffraction patterns of the nucleation layers after annealing at 1030 °C for 150 s. The layers were prepared with (a) O_2 and (b) $O_2 + H_2O$ as the oxygen precursors. The low-temperature layers deposited at 720 °C before the annealing are X-ray amorphous. The unmarked peaks belong to the c-plane sapphire substrate.

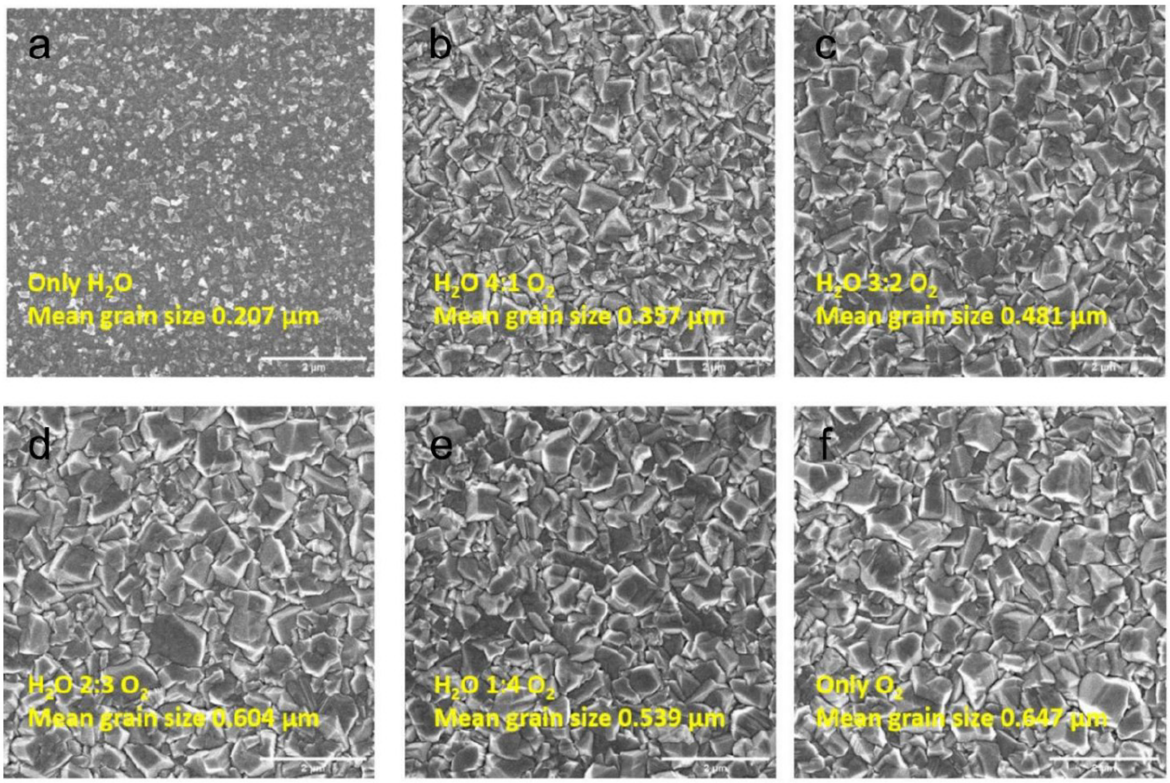

Figure S2. Scanning electron microscope images of MOCVD-grown Ga_2O_3 thin films using H_2O vapour and O_2 gas as oxygen precursors at various ratios. Total oxygen precursor flow was kept constant at 500 sccm. Higher O_2 proportion leads to larger grain sizes. Scale bars correspond to 2 μm.

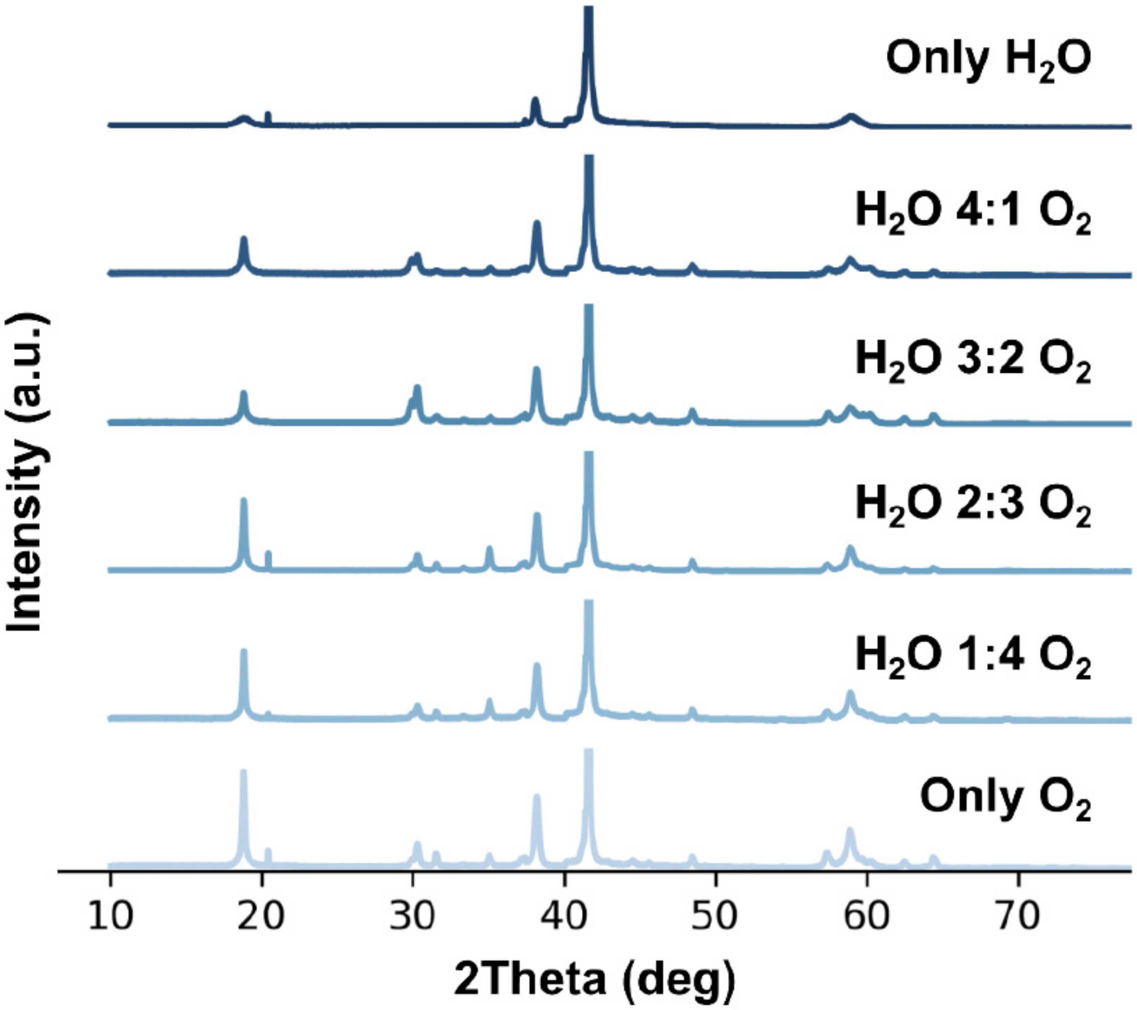

Figure S3. X-ray diffraction patterns of MOCVD-grown Ga_2O_3 thin films using H_2O vapour and O_2 gas as oxygen precursors at various ratios. Total oxygen precursor flow was kept constant at 500 sccm.

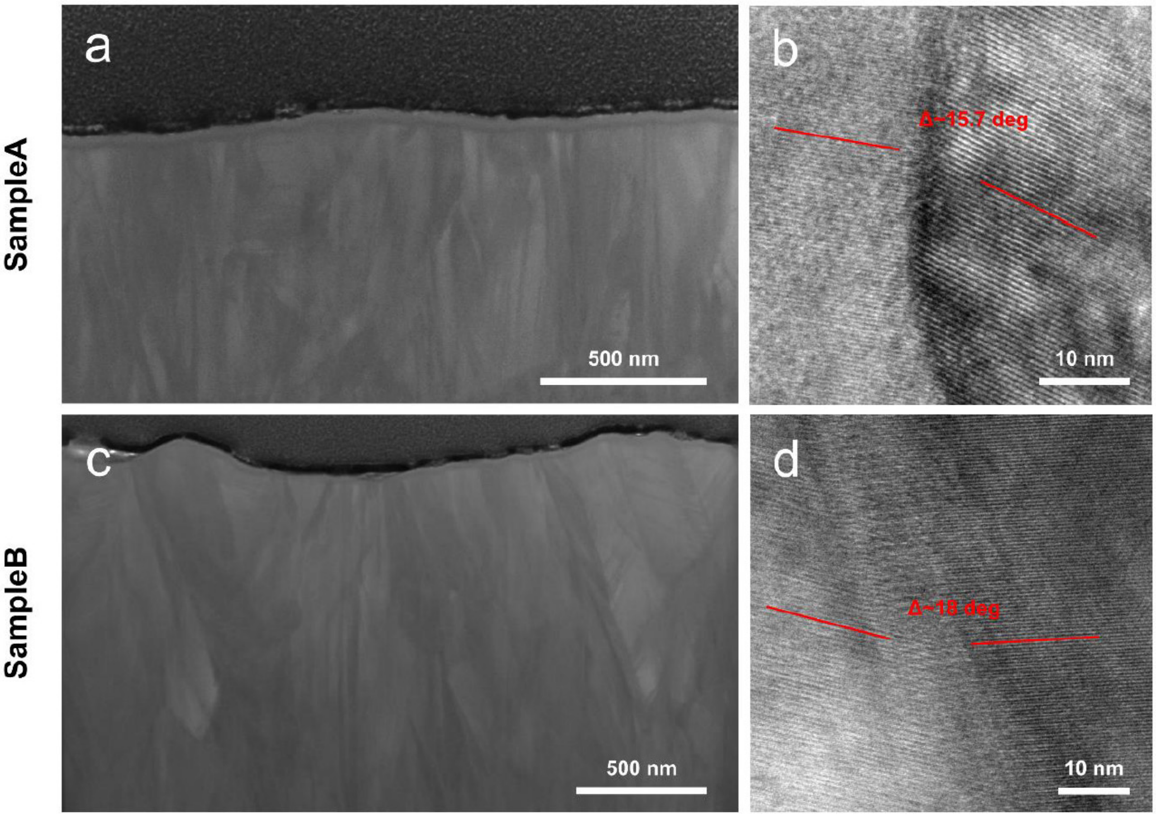

Figure S4. Scanning electron images of cross-section cuts of (a) SampleA and (c) SampleB. Transmission electron microscope images of the studied grain boundaries in (b) SampleA and (d) SampleB. Typically, most boundaries found in the samples are low-angle grain boundaries with misalignment of 1-3°, however, regions of grain misalignment up to 18° could be occasionally identified.
